# Sialic acid identity modulates host tropism of sialoglycan-binding viridans group streptococci

**DOI:** 10.1016/j.jbc.2025.110540

**Published:** 2025-07-30

**Authors:** KeAndreya M. Morrison, Rupesh Agarwal, Haley E. Stubbs, Hai Yu, Stefan Ruhl, Xi Chen, Paul M. Sullam, Barbara A. Bensing, Jeremy C. Smith, T.M. Iverson

**Affiliations:** 1 Department of Pharmacology, School of Graduate Studies, Meharry Medical College, Nashville, Tennessee, USA; 2 Oak Ridge National Laboratory, UT/ORNL Center for Molecular Biophysics, Oak Ridge, Tennessee, USA; 3 Chemical and Physical Biology Program, Vanderbilt University, Nashville, Tennessee, USA; 4 Department of Chemistry, University of California, Davis, California, USA; 5 Department of Oral Biology, University at Buffalo, The State University of New York, Buffalo, New York, USA; 6 Division of Infectious Diseases, Department of Medicine, Veterans Affairs Medical Center, University of California, San Francisco, and the Northern California Institute for Research and Education, San Francisco, California, USA; 7 Department of Biochemistry and Cellular and Molecular Biology, University of Tennessee, Knoxville, Tennessee, USA; 8 Department of Pharmacology, Vanderbilt University, Nashville, Tennessee, USA; 9 Department of Biochemistry, Vanderbilt University, Nashville, Tennessee, USA

**Keywords:** cross-reactivity, host preference, pathogenesis, commensalism, oral microbiome, infective endocarditis, sialic acid, host receptor, adhesin, Siglec-like binding region, streptococci

## Abstract

Microbial interactions with multiple species may expand the range of potential hosts, supporting both pathogen reservoirs and zoonotic spillover. Viridans group streptococci interact with host cells by engaging protein-attached glycosylations capped with terminal sialic acids (sialoglycans). One potential origin for host tropism of these streptococci arises because humans exclusively synthesize the *N*-acetylneuraminic acid (Neu5Ac) form of sialic acid, while non-human mammals synthesize both Neu5Ac and a hydroxylated *N*-glycolylneuraminic acid (Neu5Gc). However, the link between binding preference for these sialic acids and preference for the host has not been tested experimentally. Here, we investigate sialoglycan-binding by Neu5Ac/Neu5Gc cross-reactive Siglec-like binding regions (SLBRs) from two strains of streptococci, *Streptococcus gordonii* strain Challis (SLBR_Hsa_) and *Streptococcus sanguinis* strain SK36 (SLBR_SrpA_). Structural and computational analyses of SLBR_Hsa_ identified molecular details for the binding of disaccharides capped in Neu5Ac or Neu5Gc. Engineering SLBR_Hsa_ and SLBR_SrpA_ for narrow selectivity to synthetic Neu5Gc-terminated glycans shifted the binding preference from authentic human plasma receptors to plasma receptors from rat sources. However, host receptor preference did not fully recapitulate purified Neu5Ac/Neu5Gc-capped sialoglycan preference. These findings suggest that sialic acid identity modulates, but does not uniquely determine, host preference by these streptococci. This work refines our understanding of host specificity and challenges prevailing assumptions about the relative role of sialic acids in host tropism.

Viridans group streptococci are among the bacteria that can engage sialic acid-capped glycans (sialoglycans) on host cells (1). This host adherence promotes oral colonization when the sialic acid is attached to the glycans on salivary proteins, such as MUC7 (2, 3, 4). Host adherence also supports endovascular pathogenesis (5, 6). Indeed, engagement of sialoglycans attached to platelet glycoprotein Ibα (7) (GPIbα) is among the first committed steps in some bacterial endocarditis infections. This serious infection of the heart valves may result in heart failure, stroke, or permanent valve damage, even with aggressive treatment. Viridans group streptococci are responsible for 40 to 60% of cases of bacterial endocarditis (8, 9). In-hospital mortality for these infections is approximately 10%, while one- and 5-year mortality rates are estimated as 22 to 37% and 37 to 53%, respectively (10, 11, 12, 13).

Sialoglycan binding by viridans group streptococci may use serine-rich repeat (SRR) adhesins, which vary substantially in size but have modular structures that typically contain six linearly arranged functional regions (Fig. 1, *A* and *B*). These SRR adhesins are typified by two striking stretches of amino acids, SRR1 and SRR2, with serine in alternate positions (14). For example, the SRR2 of *Streptococcus sanguinis* strain SK36 contains 84 near-perfect “SAST-SASV-SAST” sequence repeats, and SRR2 of *Streptococcus gordonii* strain DL1 contains 113 repeats of the same 12 amino acids (14). Electron microscopy on SRR2 from *Streptococcus parasanguinis* suggests that it forms an extended super-coiled helix (15), allowing SRR adhesins to form long fibril-like structures that extend from the bacterial surface.

**Figure 1:**
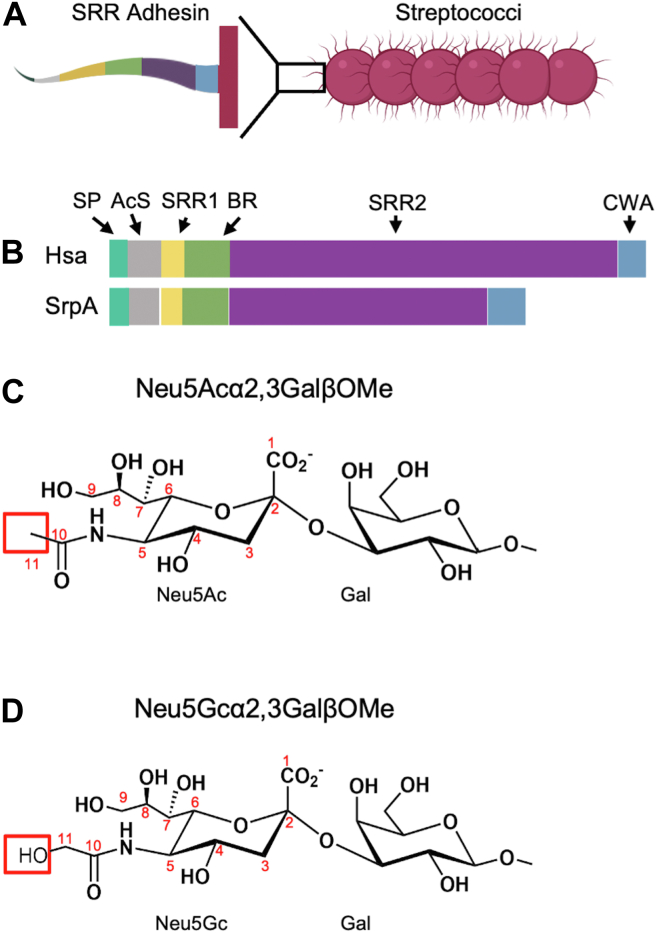
**SRR adhesins and chemical structures of sialic acid-terminated disaccharides.***A***,** Cartoon of a streptococcal bacterium showing the SRR adhesins extending from the surface. The expanded view colors the SRR by domain, illustrating a linear and modular organization. The N-terminus signal peptide (labeled SP, ∼90 amino acids, *teal*) (70) is immediately followed by an Accessory Sec targeting sequence (labeled AcS, ∼30–40 amino acids, *grey*) with these features promoting export of the adhesin (71). The third module is an SRR repeat with alternate residues being serine (SRR1, ∼50–100 amino acids, *yellow*). The fourth module is a host binding region that varies in sequence, structure, and preferred ligand (BR, commonly 200–400 amino acids, *green*). SLBRs are among the possible binding regions. A second SRR (SRR2, ∼400 to > 5000 amino acids, *purple*) is typically more regular in its sequence. The SRR adhesins terminate with an LPxTG cell wall anchoring sequence motif (labeled CWA, *blue*). *B*, To-scale schematics of the sialic acid-binding SRR adhesins for *S. gordonii* strain Challis (Hsa) and *S. sanguinis* strain SK1. Coloring is the same as *panel* A. *C*, α2-3-linked *N*-acetylneuraminic acid (Neu5Ac)-Galactose(Gal)βOMe (Neu5Acα2-3GalβOMe). The C11 is highlighted. *D*, α2-3-linked *N*-glycolylneuraminic acid (Neu5Gc)-GalβOMe (Neu5Gcα2-3GalβOMe). The additional OH linked to the C11 (OH11) is highlighted.

Of the modules within SRR adhesins, the host-binding region has received much attention. This binding region is near the N-terminus of SRR adhesins, localizing host binding to the tip of the adhesin and distal from the bacterial surface. In the sialoglycan-binding SRR adhesins, the fold of this host-binding region contains an N-terminal domain is related to that of mammalian Sialic acid-binding immunoglobulin(Ig)-like lectins (Siglecs) (16), and this Siglec-like domain directly binds host sialoglycans. All known sialoglycan-binding Siglec-like domains are associated with a C-terminal Unique domain of unknown function. These paired Siglec and Unique domains are collectively termed the Siglec-like binding region (SLBR) (16).

Both Siglec-like domains of SLBRs and Siglecs themselves are organized around a V-set Ig fold that is predominantly composed of β-strands and that has a standard nomenclature (Fig. S1). The strands of the Ig fold are designated A-G, and intervening loops are named based upon the adjacent strands (16); for example, the AB loop connects the A strand and the B strand. The sialoglycan binds above a conserved ΦTRX sequence motif on the F strand, where Φ is a hydrophobic amino acid, most commonly tyrosine.

SLBRs engage sialic-acid-capped terminal tri- and tetra-saccharides (17, 18) on heterogeneously glycosylated proteins, which can subtly differ in identity and presentation between individuals (19, 20). Perhaps surprisingly, it is difficult to predict the sialoglycan-binding spectrom of SLBRs. Even SLBRs with >90% sequence identity can exhibit a different sialoglycan-binding repertoire (18, 21). As revealed by chimeragenesis, control over the preferred sialoglycan ligand disproportionately involves three loops that surround the binding site: the CD loop, the EF loop, and the FG loop (18). Chimeragenesis revealed that these three loops control the identity of the preferred sialoglycan ligand (18). Importantly, the preferred sialoglycan ligand correlates with disease severity in an animal model, with selective binding to a sialyl-T antigen-capped receptor on GPIbα most strongly promoting endocardial infection (6).

Humans and non-human animals can differ in their production and presentation of sialoglycans. Within the context of complex glycosylations, these differences include the underlying glycan composition, the preferred glycosyl linkages, the local distribution of different sialoglycans in biological niches, and even the sialic acid itself (22, 23). Sialic acids are nine-carbon acidic sugars with more than 50 biological forms (19, 23, 24). While humans only synthesize *N*-acetylneuraminic acid (Neu5Ac, Fig. 1*C*) and its derivatives, most non-human animals can convert Neu5Ac to *N*-glycolylneuraminic acid (Neu5Gc, Fig. 1*D*) (25) and its derivatives. Neu5Ac and Neu5Gc differ by a hydroxyl group at the C11 position (26, 27) (Fig. 1*D*). Some infectious agents can bind to both forms, while others can distinguish between the two (28, 29, 30, 31). As demonstrated with glycan array analysis (21), characterized SLBRs vary in their ability to recognize purified tri- and tetrasaccharides terminating in Neu5Ac *versus* Neu5Gc, with some strongly preferring Neu5Ac and others binding to both sialic acid forms. The latter scenario is a requirement for zoonotic transmission and pathogen reservoirs. Accordingly, understanding how some streptococci bind both human and non-human forms of sialic acid can inform microbial adaptation and suggest strategies to interrupt cross-species colonization.

The two best-characterized SLBR homologs that are reported to bind with similar strength to glycosylations capped by either Neu5Ac or Neu5Gc are SLBR_Hsa_ from *S. gordonii* strain Challis (comprising residues 245–453 of the 2178 amino acid SRR) and SLBR_SrpA_ from *S. sanguinis* strain SK36 (7, 32) (comprising residues 254–444 of the 1096 amino acid SRR) (Fig. 1*B*). SLBR_Hsa_ was isolated from a human endocardial infection, and SLBR_SrpA_ was isolated from human dental plaque, but these have similar functional and structural properties. Both SLBRs bind robustly to human platelets (21, 33), SLBR_Hsa_ and SLBR_SrpA_ share 51% identity and 65% similarity (Fig. S1, Table S1), and crystal structures of unliganded SLBR_Hsa_ and SLBR_SrpA_ have an RMS deviation of 0.99 Å in their Siglec-like domain (Table S2).

Here, we use structural, computational, and molecular approaches to ask how SLBR_Hsa_ and SLBR_SrpA_ engage synthetic sialoglycans terminating in either Neu5Ac or Neu5Gc, and how this correlates with native sialoglycoside attachment. Our results reveal that Neu5Ac *versus* Neu5Gc preference modulates, but does not uniquely determine, host specificity. These findings refine our understanding of how these bacteria target their hosts, clarify molecular aspects of tropism, and may even provide initial insight into molecular drivers of bacterial species jumps.

## Results

### Structural basis for SLBR binding to Neu5Ac- and Neu5Gc-Terminated disaccharides

**Figure 2:**
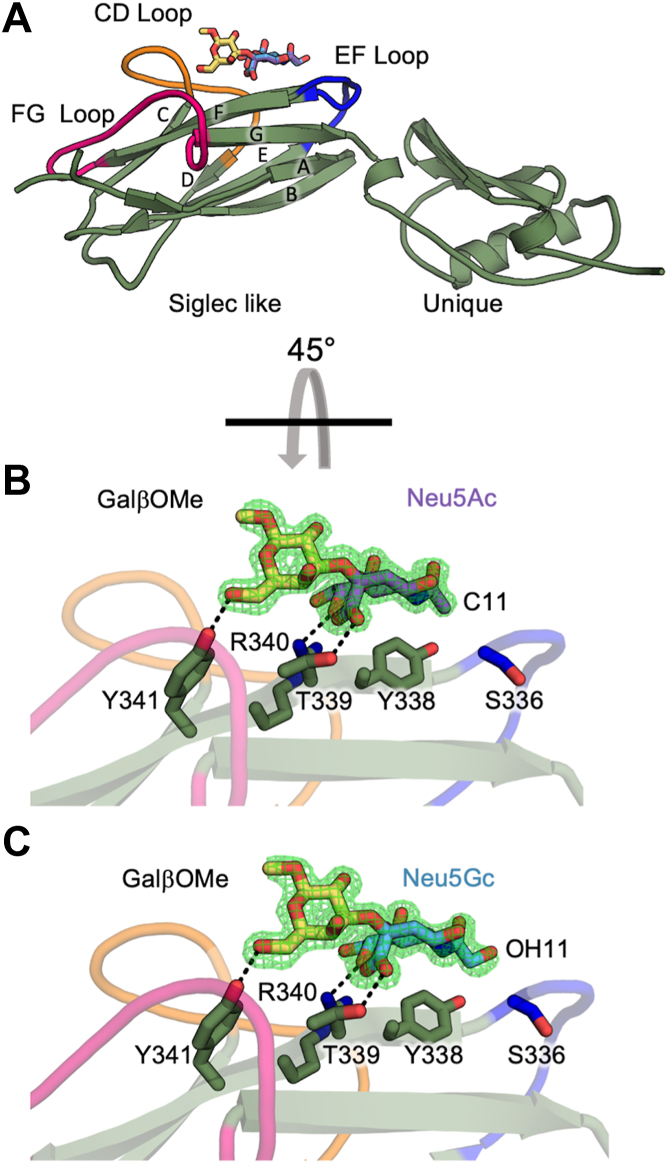
**X-ray crystal structures of Neu5Acα2-3GalβOMe or Neu5Gcα2-3GalβOMe bound to SLBR_Hsa_.***A*, *Ribbonsdiagram* of SLBR_Hsa_ with the strands of the V-set Ig fold labeled and the CD- (*orange*), EF- (*blue*), and FG-loops (*hot pink*) highlighted. Sialyl disaccharides are colored according to symbol nomenclature for glycans convention, with Neu5Ac in *purple*, Neu5Gc in *cyan*, and Gal in *yellow*. *B and C*, Zoomed-in views rotated 45° around the x-axis to highlight hydrogen bonds between *B,* Neu5Acα2-3GalβOMe or *C,* Neu5Gcα2-3GalβOMe and the ФTRX motif (SLBR_Hsa_^Y338^, SLBR_Hsa_^T339^, SLBR_Hsa_^R340^, SLBR_Hsa_^Y341^) on the F-strand. Each model is superimposed with composite omit electron density (*green mesh*).

To investigate how SLBRs engage Neu5Ac *versus* Neu5Gc, we began by determining X-ray crystal structures of SLBR_Hsa_ bound to the synthetic α2-3-linked disaccharides Neu5Acα2-3GalβOMe (SLBR_Hsa_–Neu5Gc, Fig. 2, *A* and *B*) and Neu5Gcα2-3GalβOMe (SLBR_Hsa_–Neu5Ac, Fig. 2, *A* and *C*). The SLBR_Hsa_–Neu5Gc complex was determined at 1.30 Å resolution (Fig. 2*B*, Table 1), and the SLBR_Hsa_–Neu5Ac complex was determined at 1.45 Å resolution (Fig. 2*C*, Table 1). Note that the methyl aglycon in each disaccharide (Fig. 2*B*) arises from its chemical synthesis (34, 35) and is found on a region of the glycan that does not directly contact the SLBR. Moreover, we do not observe any structural perturbations attributable to this feature here or in reported costructures of Neu5Gcα2-3GalβOMe with SLBR_SrpA_ (32, 36).

**Table 1:** X-ray crystallographic data collection and refinement statistics for SLBR_Hsa_ bound to Neu5Acα2-3GalβOMe or Neu5Gcα2-3GalβOMe

Ligand	Neu5Acα2-3GalβOMe	Neu5Gcα2-3GalβOMe
PDB ID	- 9Q5E	9Q5F
DATA ID	1020	1019
Resolution	1.30 Å	1.45 Å
Highest resolution shell	(1.32 Å–1.30 Å)	(1.48 Å–1.45 Å)
Data collection		
Beamline	SSRL 9-2	SSRL 9-2
Wavelength	0.97946 Å	0.97946 Å
Space group	P2_1_2_1_2_1_	P2_1_2_1_2_1_
Unit cell dimensions	a = 45.3 Å, b = 57.6 Å, c = 75.7 Å	a = 46.1 Å, b = 57.7 Å, c = 76.0 Å
R_sym_	0.079 (0.465)	0.086 (0.490)
R_pim_	0.023 (0.216)	0.026 (0.204)
I/σ	115.4 (1.8)	38.0 (1.8)
Completeness (%)	91.3% (46.5%)	90.4% (47.1%)
Redundancy	11.0 (4.9)	11.3 (5.9)
CC_1/2_	1.00 (0.963)	1.00 (0.964)
Refinement		
R_cryst_	0.160	0.166
R_free_	0.191	0.193
No. Mol per ASU	1	1
RMS deviation bond lengths	0.005	0.004
RMS deviation bond angles	0.843	0.752
Ramachandran Statistics		
Favored	96.5%	97.6%
Outliers	0.0%	0.0%

Values in parentheses are for the highest resolution shell. Raw data are deposited with SBGrid and can be accessed at: data.sbgrid.org/dataset/DATAID.

The positions and binding of these two sialyl disaccharide ligands are conserved between SLBR_Hsa_–Neu5Ac and SLBR_Hsa_–Neu5Gc, with superposition of the SLBR_Hsa_–Neu5Ac and SLBR_Hsa_–Neu5Gc showing that the binding position for each sialoglycan is within the error of the resolution. Each sialoglycan binds above the conserved ΦTRX motif of the F strand of the Siglec domain, where residues SLBR_Hsa_^Y338^, SLBR_Hsa_^T339^, SLBR_Hsa_^R340^, and SLBR_Hsa_^Y341^ stabilize ligands with hydrogen-bonding interactions (Fig. 2, *B* and *C*). This binding site is located between the CD, EF, and FG loops, making the overall binding mode similar to the Neu5Acα2-3Gal terminus of the sialyl tri- and tetrasaccharides in reported SLBR–sialoglycan complexes (2, 18).

Structural comparison of SLBR_Hsa_–Neu5Ac and SLBR_Hsa_–Neu5Gc with the previously reported unliganded SLBR_Hsa_ (18) also reveals similar overall folds, with RMSD values for Cα atoms of 0.157 Å (Neu5Ac-bound) and 0.158 Å (Neu5Gc-bound). Of note is the position of the EF loop. Past work shows that this EF loop can close over bound tri- and tetrasaccharides (18). In these structures, the EF loop closes over both ligands equivalently, with a maximal backbone displacement of 4.6 Å (Figs. 2, *B* and *C*, S2*A*). Loop closure is unlikely to impact Neu5Ac *versus* Neu5Gc selectivity as it does not detectably affect contacts to C11 or the C11-appended hydroxyl (OH11).

**Figure 3:**
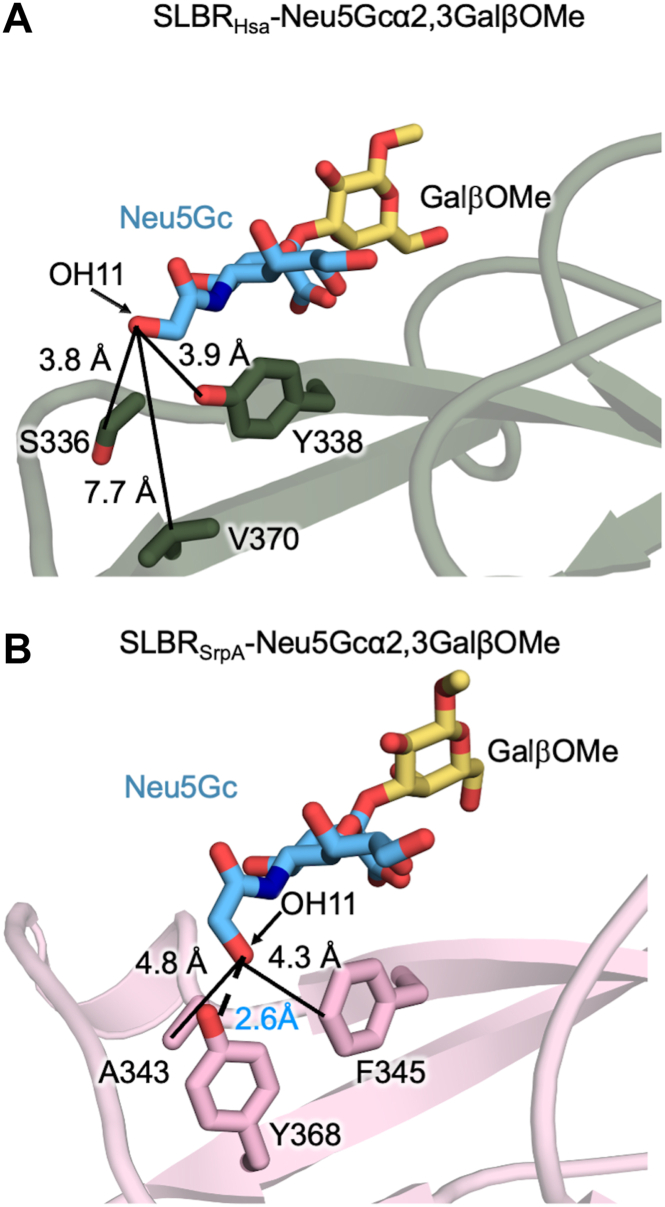
**Structures of SLBR_Hsa_–Neu5Gc and SLBR_SrpA_–Neu5Gc.** Comparison of Neu5Gc-GalβOMe binding in (*A*) SLBR_Hsa_ and (*B*) SLBR_SrpA_ (PDB 5EQ3 (36)) illustrates differences in the positioning of the C11 hydroxyl group (OH11) of Neu5Gc. *A*, In SLBR_Hsa_, the residues adjacent to sialic acid include SLBR_Hsa_^S336^ and SLBR_Hsa_^Y338^. These residues lack direct contact with the OH11 group, which is oriented away from the binding site. *B*, In contrast, SLBR_SrpA_^Y368^ hydrogen-bonds to the OH11 group.

We then compared these SLBR_Hsa_ structures to reported X-ray crystal structures of SLBR_SrpA_ with each ligand (36) (Figs. 3, S2, S3). Comparison of SLBR_Hsa_–Neu5Ac (Fig. S3*A*) with SLBR_SrpA_–Neu5Ac (36) (Fig. S3*B*) shows that Neu5Ac similarly binds above the F strand in both these SLBRs, albeit with a lateral shift in position of 1.5 Å with respect to the ΦTRX motif (Fig. S2*B*). Comparison of SLBR_Hsa_–Neu5Gc with SLBR_SrpA_–Neu5Gc (36) shows an additional difference where there is a 160° rotation of OH11 (Fig. 3, *A* and *B*). In SLBR_Hsa_–Neu5Gc, the OH11 orients toward solvent and does not interact with the protein (Fig. 3*A*). The closest protein atoms to OH11 are the SLBR_Hsa_^Y338^ side chain hydroxyl on the F-strand and SLBR_Hsa_^S336^ Oγ on the EF loop, with distances of 3.8 Å in each case. The closest atoms to C11 are the SLBR_Hsa_^Y338^ side chain hydroxyl and SLBR_Hsa_^S336^ Cβ, with distances of 3.2 Å and 3.9 Å, respectively. In comparison, a unique hydrogen bond forms between OH11 and the SLBR_SrpA_^Y368^ side chain hydroxyl that orients OH11 toward the protein (Fig. 3*B*).

**Figure 4:**
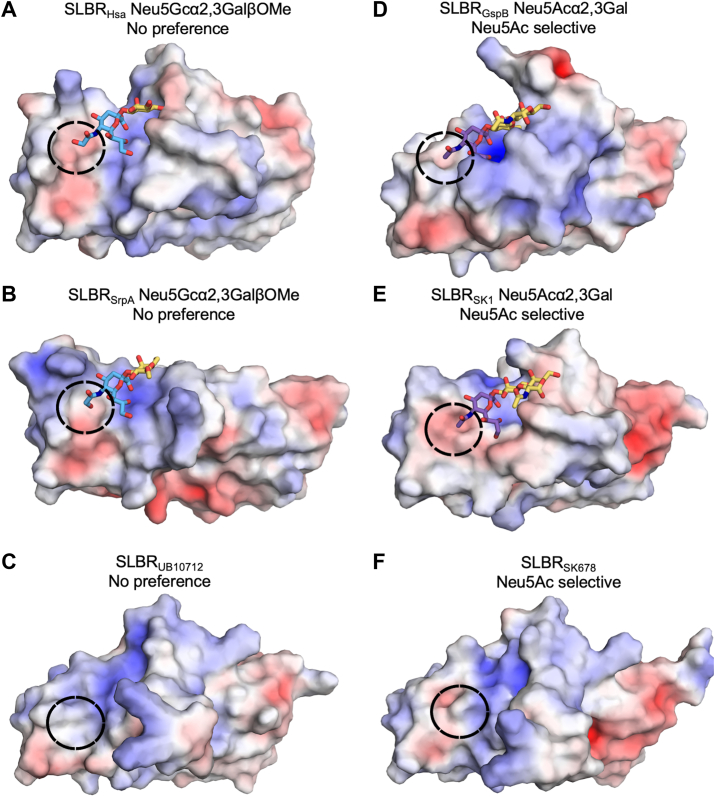
**Surface rendering of SLBR Siglec-like domains colored by electrostatic potential**. Surfaces with positive charge are colored *blue*, surfaces with negative charge are colored *red*, and neutral surfaces are colored *white*. *A*, SLBR_Hsa_. *B,* SLBR_SrpA_ (PDB 5EQ3 (36)). *C,* SLBR_UB19712_. (PDB 6EFC (18)). *D,* SLBR_GspB_ (PDB 6EFA (16)). *E,* SLBR_SK1_ (PDB 6VS7 (37)). *F,* SLBR_SK678_ (PDB 6EFI (18)). The *black circle* highlights the surface adjacent to C11/OH11 and shows a more defined binding pocket in the characterized Neu5Ac-selective SLBRs.

We next assessed whether there were differences between the Neu5Ac/Neu5Gc-binding SLBRs and Neu5Ac-selective SLBRs. We added to the analysis of SLBR_Hsa_–Neu5Ac/Neu5Gc (Fig. 4*A*, S3*A*) and SLBR_SrpA_–Neu5Ac/Neu5Gc (Figs. 4*B*, S3*B*) an additional Neu5Ac/Neu5Gc-binding comparator, SLBR_UB10712_ (Fig. 4*C*), where there is only a structure of the SLBR without ligand bound (18). For the Neu5Ac-selective SLBRs, we used SLBR_GspB_–Neu5Ac (16) (Figs. 4*D*, S3*C*), SLBR_SK1_–Neu5Ac (37) (Figs. 4*E*, S3*D*), and SLBR_SK678_ (Fig. 4*F*), which only has a structure of the SLBR without ligand bound (18). This comparison identified that the Neu5Ac/Neu5Gc-binding SLBRs have a more open binding pocket near C11 and OH11 (Fig. 4, *A*, *C* and *E*) while the Neu5Ac-selective SLBRs show a more defined pocket that likely has more precise van der Waals contacts near C11 (Fig. 4, *B*, *D* and *F*). Other potential features, such as electrostatics, bonding patterns, and water molecule substructure, did not correlate with sialic acid selectivity.

### Molecular dynamics (MD) simulations of SLBR_Hsa_ and glycan motions

To calculate whether motions in SLBR_Hsa_ might affect interactions with Neu5Ac- or Neu5Gc-terminated sialoglycans, we conducted molecular dynamics (MD) simulations. In these simulations, we separately calculated SLBR_Hsa_ flexibility and sialyl disaccharide flexibility using coordinates for Neu5Acα2-3Gal and Neu5Gcα2-3Gal that did not include the methyl aglycon.

Simulations of SLBR_Hsa_ flexibility were initiated from the unliganded conformation of SLBR_H__sa_ (18), allowing the loops to equilibrate around each ligand without pre-imposed bias. As in prior simulations (18), the CD-, EF-, and FG-loops adjacent to the binding pocket exhibited the greatest flexibility, supported by both root-mean-square fluctuation (RMSF) (Fig. S4) and crystallographic temperature factor analyses (Fig. S5). In addition, the EF loop of SLBR_Hsa_ closed over both Neu5Acα2-3Gal and Neu5Gcα2-3Gal, allowing the SLBR_Hsa_^K335^ backbone carbonyl to approach the O4 atom of each sialoglycan (Fig. S6). We particularly evaluated SLBR_Hsa_^V370^ on the G strand, which is analogous to the SLBR_SrpA_^Y368^ that hydrogen-bonds with Neu5Gc OH11. SLBR_Hsa_^V370^ did not exhibit flexibility or approach either sialoglycan. In fact, no protein atoms came within 3 Å of the Neu5Ac/Neu5Gc C11 or Neu5Gc OH11 at any point during the simulation. These predictions suggest that, on the timescale of the simulation, loop motion in SLBR_Hsa_ does not form stable direct contacts with C11 or OH11.

We designed a second set of calculations to evaluate whether sialoglycan flexibility might contribute to transient interactions with SLBR_Hsa_ by allowing C11, OH11, or other atoms to approach the protein surface. These simulations were initiated with the EF loop of SLBR_Hsa_ in the closed conformation around each disaccharide. Throughout the simulations, the Neu5Acα2-3Gal and Neu5Gcα2-3Gal disaccharides remained stably bound with a conserved orientation. The sialic acid and galactose moieties showed minimal positional fluctuation. In the Neu5Gc-bound simulation, the OH11 did not form persistent contacts with the protein surface. These findings are consistent with the crystal structure of SLBR_Hsa_–Neu5Gc, in which OH11 is rotated away from the protein and SLBR_Hsa_ does not directly engage Neu5Gc OH11 (Fig. 3). The replicates showed a narrow probability distribution around the pose of the crystal structure (Fig. S7). The mean RMSD is 0.85 Å (±0.14 Å) for Neu5Ac and 0.83 Å (±0.15 Å) for Neu5Gc.

### Binding of SLBR_Hsa_^V370^ and SLBR_SrpA_^Y368^ mutants to synthetic disaccharides and plasma glycoproteins

**Figure 5:**
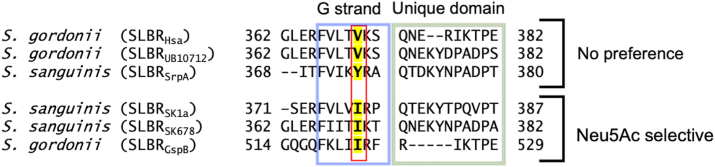
**Structure-based sequence alignment of SLBRs with reported Neu5Ac/Neu5Gc binding data.** PDB files are from SLBR_Hsa_ (PDB 6EFC (18)), SLBR_SrpA_ (PDB 5EQ3 (36)), SLBR_UB19712_ (PDB 6EFC (18)), SLBR_GspB_ (PDB 6EFA (16)), SLBR_SK1_ (PDB 6VS7 (37)), and SLBR_SK678_ (PDB 6EFI (18)). Sequences are from WP_081102781.1 from *S. gordonii* strain Challis (SLBR_Hsa_) (14, 18), WP_045635027.1 from *S. gordonii* strain UB10712 (18, 58), WP_011836739.1 from *S. sanguinis* strain SK36 (SLBR_SrpA_) (36, 59), WP_080555651.1 from *S. sanguinis* strain SK1 (SLBR_SK1a_ and SLBR_SK1b_) (37, 60), WP_125444035.1 from *S. sanguinis* strain SK678 (18, 60), and WP_125444382.1 from *S. gordonii* strain M99 (SLBR_GspB_) (16, 61). Full sequence alignment is in Figure S1. The highlighted residue is mutated in this study. Sequence identity/similarity and RMSD values are in Tables S1 and S2.

We next explored indirect influences that might help SLBRs distinguish between these two forms of sialic acid. We focused on SLBR_Hsa_^V370^ and SLBR_SrpA_^Y368^. Among characterized SLBRs, residues at the equivalent position are typically hydrophobic: Val, Ile, Tyr, or Phe (Fig. 5, Fig. S1). SLBR_Hsa_^V370^ does not directly contact the sialic acid, and the closest side chain atoms are 7.5 Å away from C11 (Figs. 3*A*, S3*A*). In addition, SLBR_Hsa_^S336^ and SLBR_Hsa_^Y338^ are located between the SLBR_Hsa_^V370^ side chain and C11, blocking the possibility of direct contact. However, the analogous SLBR_SrpA_^Y368^ forms a hydrogen bond with the OH11 group of Neu5Gc *via* its side chain hydroxyl (Fig. 3*B*). The Cα of SLBR_SrpA_^Y368^ is 7.6 Å from OH11, and the close approach of the side chain hydroxyl is due to the length of the residue.

**Figure 6:**
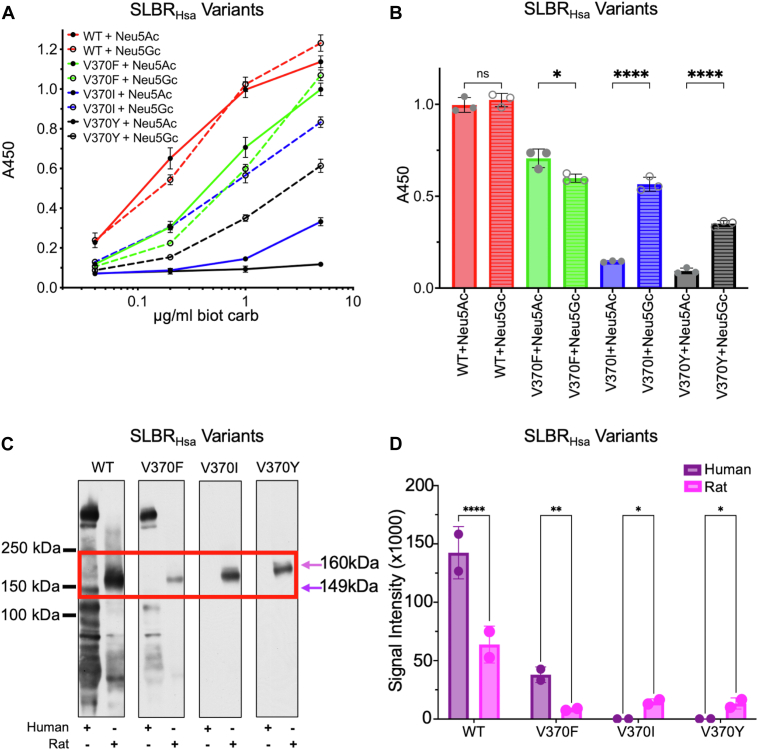
**Wild-type and mutant SLBR_Hsa_ binding to purified biotinylated disaccharides far Western analysis of binding to human or rat plasma.***A*, ELISA curves for wildtype and mutant GST-tagged SLBR_Hsa_ (GST-SLBR_Hsa_) binding to biotinylated Neu5Ac- and Neu5Gc-terminated disaccharides at the indicated concentrations. Measurements were performed using 500 nM of immobilized GST-SLBR_Hsa_, and the indicated concentrations of each ligand are shown as the mean ± SD. (n = 3 independent experiments performed on protein from a single preparation). *B*, Comparison of the levels of binding of wildtype and mutant GST-SLBR_Hsa_ at a concentration of 1 μg/ml biotinylated disaccharides. *p*-values for Neu5Ac *versus* Neu5Gc binding to SLBR_Hsa_ are: SLBR_Hsa_^WT^*p* = 0.9585, SLBR_Hsa_^V370F^*p* = 0.0121, SLBR_Hsa_^V370I^*p ≤* 0.0001, SLBR_Hsa_^V370Y^*p ≤* 0.0001, as evaluated by one-way ANOVA. *C*, Far-Western blot of wild-type and mutant GST-SLBR_Hsa_ against plasma glycoproteins. Glycoprotein loads were standardized by assessing total protein prior to electrophoretic separation through a 3 to 8% polyacrylamide gradient. No signals were detected outside of the cropped region. As previously identified (6), the proteins highlighted by the *red box* are human GPIbα (149 kDa, *purple arrow*) or rat GPIbα (160 kDa, *pink arrow*). Uncropped blots, including replicates, are in Figure S8. *D*, Dosimetry of blots showing absolute binding, without regard to molecular weight, measured with IMAGEJ version 1.54 (72). *p*-values for human *versus* rat binding to SLBR_Hsa_ are: SLBR_Hsa_^WT^*p ≤* 0.0001, SLBR_Hsa_^V370F^*p* = 0.0012, SLBR_Hsa_^V370I^*p* = 0.0155, SLBR_Hsa_^V370Y^*p* = 0.0214, as evaluated by two-way ANOVA.

**Figure 7:**
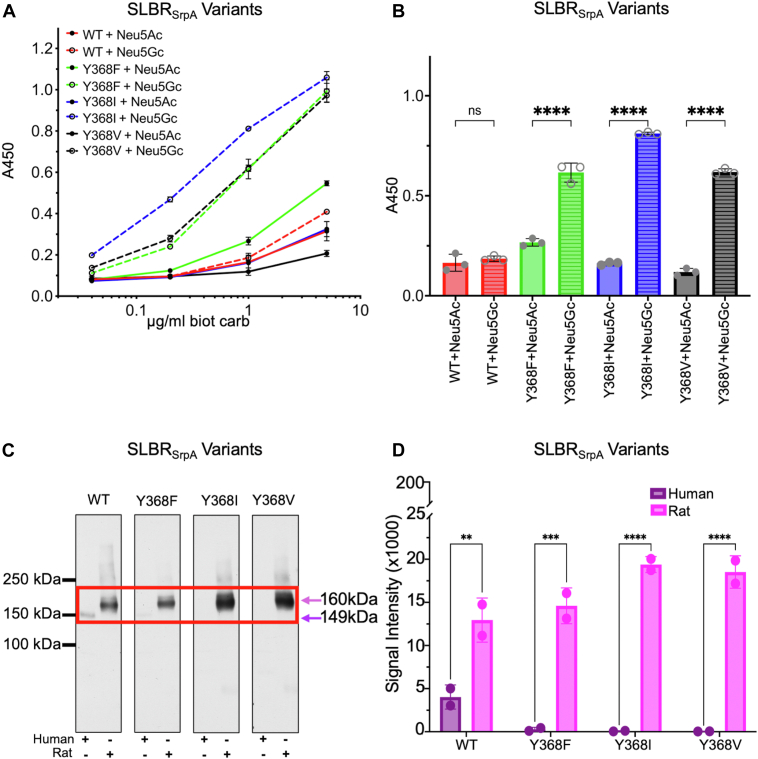
**Wild-type and mutant SLBR_SrpA_ binding to purified and biotinylated disaccharides and far Western analysis of binding to human or rat plasma.***A*, ELISA curves for wildtype and mutant GST-tagged SLBR_SrpA_ (GST-SLBR_SrpA_) binding to biotinylated Neu5Ac- and Neu5Gc-terminated disaccharides at the indicated concentrations. Measurements were performed using 500 nM of immobilized GST-SLBR, and the indicated concentrations of each ligand are shown as the mean ± SD (n = 3 independent experiments performed on protein from a single preparation). *B*, Comparison of the levels of wildtype and mutant GST-SLBR_SrpA_ binding to 1 μg/ml biotinylated Neu5Ac *versus* Neu5Gc disaccharides. *p*-values for Neu5Ac *versus* Neu5Gc binding to SLBR_SrpA_ are: SLBR_SrpA_^WT^*p* = 0.9407, SLBR_SrpA_^Y368F^*p ≤* 0.0001, SLBR_SrpA_^Y368I^*p ≤* 0.0001, SLBR_SrpA_^Y368V^*p ≤* 0.0001, as evaluated by one-way ANOVA, *C*, Far-Western blot of wild-type and mutant GST-SLBR_SrpA_ against plasma glycoproteins. Glycoprotein loads were standardized by assessing total protein prior to electrophoretic separation through a 3 to 8% polyacrylamide gradient. No signals were detected outside of the cropped region. The proteins in the *red box* are human GPIbα (149 kDa, *purple arrows*) or rat (160 kDa, *pink arrows*) GPIbα^6^. Uncropped blots, including replicates, are in Figure S8. *D*, Dosimetry of blots showing absolute binding, without regard to molecular weight, measured with IMAGEJ version 1.54 (72). *p*-values for human *versus* rat binding to SLBR_SrpA_ are: SLBR_SrpA_^WT^*p* = 0.0011, SLBR_SrpA_^Y368F^*p* = 0.0002, SLBR_SrpA_^Y368I^*p ≤* 0.0001, SLBR_SrpA_^Y368V^*p ≤* 0.0001, as evaluated by two-way ANOVA.

To investigate whether residues at this position play an allosteric role in distinguishing between sialic acid forms, we made substitutions in SLBR_Hsa_ (Fig. 6) and SLBR_SrpA_ (Fig. 7). SLBR_Hsa_^V370^ was mutated to Ile, Phe, or Tyr, and SLBR_SrpA_^Y368^ was mutated to Val, Ile, or Phe, so that each of these SLBR scaffolds had a version with Val, Ile, Tyr, or Phe at this position. We assessed binding to purified and biotinylated Neu5Acα2-3Gal or Neu5Gcα2-3Gal by ELISA (Figs. 6, *A* and *B*, 7, A and B) and to human and rat plasma glycoproteins (Figs 6, *C* and *D*, 7, C and D, S8), evaluating both the overall level of binding, the preference for Neu5Ac- *versus* Neu5Gc-terminated disaccharides, and the preference for human *versus* rat plasma glycoproteins.

Purified disaccharides are partial ligands. SLBRs are therefore expected to have lower affinity for disaccharides than for a host glycoprotein. While SLBRs bind disaccharides with lower affinity than they bind native glycoproteins, these partial ligands are ideal for dissecting the contributions of sialic acid identity to binding preference because they contain the minimal unit shown to bind all characterized SLBRs. In contrast, SLBRs bind to native sialoglycans with higher affinity, but the binding is influenced by the identity and linkages of the underlying sialoglycosides. For the purified disaccharides, as compared to wild-type SLBR_Hsa_, SLBR_Hsa_^V370I^ and SLBR_Hsa_^V370Y^ exhibited reduced but statistically significant Neu5Acα2-3Gal binding, while SLBR_Hsa_^V370Y^ lacked statistically significant binding under the conditions tested (Fig. 6, *A* and *B*). In contrast, all SLBR_Hsa_ proteins retained binding to Neu5Gcα2-3Gal, although overall levels were moderately reduced. Because of the disproportionate loss of Neu5Acα2-3Gal binding, all SLBR_Hsa_ mutants became more selective for Neu5Gc-terminated disaccharides.

Mutations in SLBR_SrpA_ produced an even more striking result. All variants showed substantially increased relative binding to Neu5Gc-termined disaccharides when compared to the wild-type SLBR_SrpA_. The strongest effect was observed with the SLBR_SrpA_^Y368I^, where binding to Neu5Gcα2-3Gal increased 5.1-fold when 1 μg/ml of each biotinylated disaccharide was used (Fig. 7, *A* and *B*). The SLBR_SrpA_^Y368V^ (3.9-fold increase) and SLBR_SrpA_^Y368F^ (3.9-fold increase) mutants also showed substantial increases in Neu5Gcα2-3Gal binding. At the same time, Neu5Acα2-3Gal binding increased 2.7-fold in SLBR_SrpA_^Y368F^ but was statistically identical in the remaining mutants. Because of the very large gains in Neu5Gcα2-3Gal binding, SLBR_SrpA_ mutants were even more strongly selective for Neu5Gc-terminated disaccharides than were SLBR_Hsa_ mutants.

We next investigated binding to authentic glycoproteins from human and rat sources by far Western analysis. Within the limits of detection, human plasma contains glycoproteins that only terminate in Neu5Ac and its derivatives, while rats have glycoproteins terminating in both Neu5Ac and Neu5Gc (25). Contrary to what was observed in ELISAs with synthetic disaccharides (Fig. 6, *A* and *B*), wild-type SLBR_Hsa_ showed a robust interaction with the human plasma glycoproteins and a substantial preference for binding to glycoproteins within human plasma (Fig. 6, *C* and *D*). However, there are some curious nuances. Of particular note is binding to the ∼150 kDa GPIbα glycoprotein implicated as the receptor in endocardial infection (3). SLBR_Hsa_ exhibits more total binding to human plasma glycoproteins than rat plasma glycoproteins, but much of this is off target with respect to GPIbα. While there is less total binding of SLBR_Hsa_ to rat plasma glycoproteins, this SLBR more selectively recognizes rat GPIbα.

Among SLBR_Hsa_ mutants, SLBR_Hsa_^V370F^ retained a preference for human plasma glycoproteins, albeit at a three-fold decrease in total binding (Fig. 6, *C* and *D*). SLBR_Hsa_^V370F^ also lost detectable binding to human GPIbα and only bound to off-target glycoproteins not associated with endocarditis. All detectable binding of SLBR_Hsa_^V370F^ to rat plasma glycoproteins is to GPIbα. The remaining two SLBR_Hsa_ mutants only detectably bound to rat GPIbα.

Wild-type SLBR_SrpA_ bound more robustly to rat plasma (Fig. 7, *C* and *D*), again in contrast to the statistically identical binding to Neu5Ac- and Neu5Gc-terminated synthetic disaccharides in ELISA (Fig. 7, *A* and *B*). Moreover, almost all detectable SLBR_SrpA_ binding was to GPIbα in both human and rat plasma (Fig. 7, *C* and *D*). Of the SLBR_SrpA_ mutants, SLBR_SrpA_^Y368V^ and SLBR_SrpA_^Y368I^ either had statistically identical or modestly increased binding to rat GPIbα, which contrasted with the large increase of binding of each of these SLBR mutants to purified Neu5Gc-terminated disaccharides. For example, wild-type SLBR_SrpA_ and SLBR_SrpA_^Y368F^ had statistically identical binding to rat GPIbα, even though SLBR_SrpA_^Y368F^ had five-fold greater binding to synthetic Neu5Gc-terminated disaccharides in ELISA. The SLBR_SrpA_^Y368F^ mutant showed barely detectable binding to human plasma glycoproteins, despite a ∼3-fold increase in binding to Neu5Acα2-3Gal binding in the ELISA assays. The remaining two SLBR_SrpA_ mutants had undetectable binding to human plasma glycoproteins under these conditions, and all SLBR_SrpA_ mutants substantially shifted the binding preference toward rat glycoproteins.

Taken together, our mutational analysis identified that the SLBR_Hsa_^V370^ and SLBR_SrpA_^Y368^ equivalent position affects both sialic acid selectivity and host preference. All tested mutations at this position increased Neu5Gc selectivity and increased preference for rat plasma glycoproteins, even when the substitutions maintained hydrophobicity near the sialoglycan binding site and might be expected to repel the more hydrophilic Neu5Gc. Despite this trend, binding preference for Neu5Ac *versus* Neu5Gc-capped purified glycans does not fully correlate with binding to human *versus* rat glycoproteins and suggests that sialic acid identity is a secondary determinant of host preference.

## Discussion

Our data provide insight into how SLBRs from viridans group streptococci engage Neu5Ac- and Neu5Gc-terminated sialoglycans. We define the structural basis for the binding of SLBR_Hsa_ to the Neu5Acα2-3Gal and Neu5Gcα2-3Gal sialyl disaccharides and demonstrate that Neu5Ac/Neu5Gc preference can be modified through mutation. Furthermore, we show that binding to synthetic Neu5Ac- and Neu5Gc-terminated disaccharides only partially predicts binding to authentic human and rat plasma sialoglycoproteins. Intriguingly, SLBR mutants also showed narrowed glycoprotein engagement, losing off-target binding and very selectively engaging the GPIbα glycoprotein, where the interaction is implicated in infective endocarditis (7, 38, 39). Several aspects of these results suggest important nuances in how SLBRs mediate host glycoprotein recognition.

A key finding was that Neu5Ac/Neu5Gc selectivity appears to be modulated by indirect effects rather than direct contacts (Figs. 2 and 3). In addition, there was no evidence that flexibility contributes meaningfully to Neu5Ac/Neu5Gc cross-reactivity (Figs. S4 and S5). This contrasts with the molecular basis for SLBR selectivity for Neu5Ac-terminated tri- and tetrasaccharide sialoglycans, in which binding preferences are largely dictated by direct SLBR-ligand interactions, as assisted by protein flexibility (18). In those cases, chimeragenesis and point mutagenesis resulted in predictable changes to the sialoglycan binding repertoire (18). The same study identified that the flexible CD-, EF-, and FG-loops (Fig. 2*A*) contribute to broad selectivity. Flexibility of the EF-loop was particularly important for allowing the SLBR to adjust in response to ligand structure, enabling reactivity with structurally diverse Neu5Ac-capped α2-3-linked glycans (18).

Another striking finding was the consistent gain in Neu5Gc preference for both SLBRs following point mutagenesis (Figs. 6 and 7). This asymmetry may arise from differences in the requirements for each form of sialic to interact with SLBRs (Figure 2, Figure 3, Figure 4). A polar OH11 of Neu5Gc may benefit from a more open binding pocket (Fig. 4, *A*–*C*) to allow local motions and enhanced solvent exposure. This may be recapitulated by perturbed local packing that inevitably accompanies mutagenesis. The more hydrophobic Neu5Ac (40) may require more precise packing to desolvate the C11 methyl group (Fig. 4, *D*–*F*). Despite this, all naturally occurring SLBRs that have been experimentally characterized either exhibit a strong binding preference toward sialoglycans terminating in Neu5Ac (2, 18, 21) or bind equivalently to sialoglycans terminating in Neu5Ac or Neu5Gc (2) (Figs. 6 and 7). The absence of characterized SLBRs that prefer Neu5Gc-terminated sialoglycans may reflect sample bias because the library of available viridans group streptococci favors bacteria isolated from humans (2, 3, 4). These isolates would have evolved in the context of selective pressure to engage Neu5Ac-terminated sialoglycosides of humans (5, 6).

These observations extend our understanding of the host tropism of sialoglycan-binding pathogens. One hypothesis for the divergence in sialic acid composition between humans and non-human animals involves the evolutionary response to host-pathogen interplay. Non-human animals synthesize CMP-linked Neu5Gc when CMP-Neu5Ac hydroxylase hydroxylates the *N*-acetyl group (*i.e.,* C11) of CMP-Neu5Ac (Fig. 1, *C* and *D*). Our study used samples from rats, where Neu5Gc has been experimentally measured at different levels depending on the biological location. The ratio in developing lungs is measured as ∼75% Neu5Ac and ∼25% Neu5Gc (25), while the level in rat GPIbα extract is measured as ∼28% Neu5Ac and ∼58% Neu5Gc (6). Humans have an inactive CMP-Neu5Ac hydroxylase enzyme due to a loss-of-function mutation occurring approximately two million years ago (23, 27, 41, 42), and therefore only synthesize Neu5Ac. It has been postulated that this human loss-of-function mutation conferred protection against ancestral forms of *Plasmodium*, the parasitic protozoan responsible for malaria (43), as some modern *Plasmodium falciparum* strains rely primarily on sialic acid for invasion (44, 45).

However, the relationship between pathogenesis and sialic acid identity is not straightforward. In many cases, the linkage specificity (*e.g.*, α2-3 vs α2-6 of C6 to the underlying Gal) plays a larger role than sialic acid identity in determining virulence. One example is influenza, which binds to human sialoglycans through hemagglutinins. The H1 and H3 hemagglutinins specifically bind α2-6-linked sialic acids, which are abundant in mammalian airways (46). These strains are infectious to humans and other mammals. By contrast, H5 hemagglutinin found in strains of the avian flu binds to α2-3-linked sialic acids, which are abundant in avian airways but rare in mammalian airways (47, 48). The H5N1 avian flu has a high mortality rate in both mammals and birds, but H5N1 strains are substantially less infective for humans at the present time (49). Intriguingly, Neu5Gc-terminated sialoglycans can act as decoy receptors for some strains of influenza, where they support hemagglutinin binding but inhibit viral entry (35, 36, 37). Coronaviruses such as SARS-CoV and SARS-CoV-2 can also engage host sialoglycans (50, 51, 52), and this interaction synergizes with binding to ACE2 to promote viral entry (50, 51, 52). Although this finding is quite recent, early work suggests narrow linkage selectivity for this interaction (50, 51, 52).

Our results suggest that the host tropism of viridans group streptococci blends Neu5Ac/Neu5Gc selectivity with a broader glycan context. Host glycoprotein engagement is certainly influenced by the ability to bind Neu5Ac *versus* Neu5Gc-capped sialoglycans, as evidenced by shifts in SLBR binding toward rat glycoproteins as Neu5Gc preference increased (Figs. 6 and 7). However, SLBR interactions with synthetic sialyl disaccharides did not fully predict binding to authentic plasma glycoproteins, which can have larger, more complex underlying glycan structures. This highlights a secondary role for sialic acid identity relative to other determinants, such as glycan linkage and glycoprotein presentation. The discrepancy between plasma glycoprotein binding and synthetic sialyl disaccharide recognition is unlikely to result from alternative glycoprotein engagement. The only other sialic acid derivative present in humans is 2-keto-3-deoxy-D-glycero-D-galactonononic acid (Kdn), which accounts for less than 1% of human glycans and is not measurably incorporated into glycoproteins or glycolipids presented on the surface of human cells (53, 54). Recent studies show that Kdn can be incorporated into bacterial glycoconjugates under experimental conditions (54), reinforcing the limited relevance of Kdn as an SLBR target in the host. Additionally, we are not aware of any literature suggesting that SLBRs could engage non-sialoglycoside glycoproteins.

Together, our data support a model in which Neu5Ac/Neu5Gc preference modulates, but does not uniquely determine, SLBR-mediated host glycoprotein recognition by viridans group streptococci. By uncovering distinct structural and allosteric mechanisms of sialoglycan recognition, this work extends our understanding of SLBR specificity and host range, offering insight into bacterial adaptation. Finally, our work identifies that models of host tropism will benefit from a nuanced view of glycoprotein chemistry that encompasses sialic acid identity, glycan context, and glycan presentation.

## Experimental procedures

### Protein expression and purification for X-ray crystallography

SLBR_Hsa_ was expressed and purified as previously described (18). Briefly, the pSV278 vector, created at Vanderbilt University, is a pET27 derivative that appends a thrombin cleavable His-maltose-binding protein (MBP) tag at the N-terminus of the protein of interest, where the MBP is from pMAL. This low-copy-number plasmid encodes kanamycin resistance and places the gene of interest under the control of the T7 *lac* promoter. Using pSV278, His_6_-MBP-SLBR_Hsa_ was expressed in *Escherichia coli* BL21 (DE3) in Terrific Broth medium supplemented with 50 μg/ml kanamycin at 37 °C. At an OD_600_ of 1.0, the temperature was lowered to 24 °C, and expression was induced with 1 mM isopropyl β-d-thiogalactopyranoside (IPTG) for 6 h. Cells were harvested by centrifugation at 5000*g* for 15 min, washed with 0.1 M Tris-HCl, pH 7.5, and stored at −20 °C before purification.

Frozen cells were resuspended in buffer containing 20 to 50 mM Tris-HCl, pH 7.5, 150 to 200 mM NaCl, 1 mM EDTA, 1 mM PMSF, 2 μg/ml Leupeptin, 2 μg/ml Pepstatin, then disrupted by sonication. Lysate was clarified by centrifugation at 38,500*g* for 35 to 60 min and passed through a 0.45 μm filter. Purification of His_6_-MBP-SLBR_Hsa_ was performed at 4 °C with an MBP-Trap column and eluted in 10 mM maltose. Eluted proteins were concentrated in a 10 kDa molecular weight cutoff concentrator and exchanged into buffer containing 20 mM Tris-HCl, pH 7.5, and 200 mM NaCl. The His_6_-MBP affinity tags were cleaved from SLBR_Hsa_ with 1 U of thrombin per mg of His_6_-MBP-SLBR_Hsa_ overnight at 4 °C. The cleaved His_6_-MBP tag was separated from pure SLBR_Hsa_ using a Superdex 200 Increase 10/30 Gl column equilibrated in 20 mM Tris-HCl, pH 7.5, and 200 mM NaCl. After purification, the protein was > 95% pure, as assessed by SDS-PAGE, and was stored at −80 °C.

### Crystallization and collection of X-ray diffraction data

Crystals of SLBR_Hsa_ (21.6 mg/ml in 20 mM Tris-HCl, pH 7.2) were formed by the sitting drop vapor diffusion method by equilibrating 1 μl protein and 2 μl reservoir solution over 50 μl of reservoir solution. Reservoir solution contained 0.1 M SPG buffer pH 10.0 (Hampton Research), and 25% PEG 3350. The SPG buffer is a mixture of succinic acid, sodium phosphate monobasic monohydrate, and glycine, with a molar ratio of 2:7:7. The 1 M stock solution consists of 0.125 M succinic acid, 0.438 M sodium phosphate monobasic monohydrate, and 0.437 M glycine. The buffer is titrated to pH 10.0 at 25 °C using sodium hydroxide. Co-crystals of SLBR_Hsa_ with sialoglycan ligands were prepared by soaking fully formed crystals in reservoir solution supplemented with 5 mM of each ligand for 20 h. Crystals did not require cryoprotection beyond the reservoir solution and were cryocooled by plunging into liquid nitrogen. Data were collected at −180 °C on beamline 9-2 at the Stanford Synchrotron Radiation Lightsource. Data were processed in HKL2000 (55).

### Structure determination and refinement

The structure of each sialoglycan-bound SLBR_Hsa_ was determined using isomorphous replacement by removing all solvent molecules from unliganded SLBR_Hsa_ (PDB entry 6EFC (18)) and performing rigid body refinement in PHENIX (56). The resultant model was improved using alternate rounds of model building in COOT (57) and refinement in PHENIX (56). Throughout the process, the R_free_ reflections were selected to be the same as for the unliganded SLBR_Hsa_ (PDB entry 6EFC (18)). Data collection and refinement statistics are listed in Table 1.

### Structure-based sequence alignment

Structure-based sequence alignment of SLBRs was performed in COOT (57) by superimposing resected Siglec domains from crystal structures of representative SLBRs. Sequences are from WP_081102781.1 from *S. gordonii* strain Challis (SLBR_Hsa_, PDB entry 6EFC) (14, 18), WP_045635027.1 from *S. gordonii* strain UB10712 (PDB entry 6EFF) (18, 58), WP_011836739.1 from *S. sanguinis* strain SK36 (SLBR_SrpA_, PDB entry 5EQ2) (36, 59), WP_080555651.1 from *S. sanguinis* strain SK1 (SLBR_SK1a_ and SLBR_SK1b_, PDB entry 6VS7) (37, 60), WP_125444035.1 from *S. sanguinis* strain SK678 (PDB entry 6EFI) (18, 60), and WP_125444382.1 from *S. gordonii* strain M99 (SLBR_GspB_, PDB entry 6EFA) (16, 18, 61).

### Sialoglycan reagents

Neu5Acα2-3GalβOMe and Neu5Gcα2-3GalβOMe used in crystallography studies were prepared as previously reported (32, 36). Biotinylated sialyl disaccharides for the ELISAs were purchased from Sigma (GNZ-0035-BM for Neu5Ac and GNZ-0018-BM for Neu5Gc).

### MD simulations

The crystal structures of SLBR_Hsa_ bound to either Neu5Acα2-3Gal or Neu5Gcα2-3Gal were used to generate starting models of the Siglec-like domains for MD simulations. MD was performed on the proteins and glycan ligands using the Amber14 ff14SB (62) and Glycam06 (63) force fields, respectively, with a non-bonded cutoff of 10 Å using the Particle Mesh Ewald algorithm (64). Each protein-glycan system was hydrated by water model TIP3P (65) using an octahedral box of 10 Å around the protein in each direction. Initially, the protein was held fixed with a force constant of 500 kcal mol^−1^ Å^−2^, while the system was energy minimized with 500 steps of steepest descent. This was followed by 500 steps of energy minimization with the conjugate gradient method. In a second minimization step, the restraints on the protein were removed, and 1000 steps of steepest descent minimization were performed, followed by 1500 steps of conjugate gradient. The system was heated to 300 K while holding the protein fixed with a force constant of 10 kcal mol^−1^ Å^−2^ for 1000 steps. Then, the restraints were removed, and 1000 MD steps were performed. The SHAKE algorithm (66) was used to constrain all bonds involving hydrogen in the simulations. MD production runs were performed at 300 K using the NPT ensemble and a 2-fs time step. The temperature was fixed with the Langevin dynamics thermostat (67), and the pressure was fixed with the Monte Carlo barostat (68). Three independent runs were performed for each simulation. All analyses were done using the Pytraj package (69).

### Protein expression and purification for ELISAs and far Western blotting

SLBR_Hsa_, SLBR_SrpA_, and all variants were expressed and purified as previously described (18). Point mutants were created from already cloned SLBRs in vector pBG101 (Vanderbilt University), which encodes a His_6_-Glutathione-S-transferase (GST) tag at the N-terminus, followed by a 3C protease cleavage site. His_6_-GST-SLBRs were expressed in *E. coli* BL21 (DE3) in Miller's Luria Broth at 37 °C with 50ug/ml of kanamycin for ∼3 h to reach an A_600_ of 0.80. SLBR expression was induced with 1 mM IPTG for 3 h at 24 °C. Cells were harvested by centrifugation at 7000 *g* for 20 min. Cell pellets were resuspended in 125 mM Tris, 150 mM sodium chloride, pH 8.0, supplemented with 1 mM EDTA, 1 mM PMSF, 2 μg/ml Leupeptin, 2 μg/ml pepstatin, then lysed by sonication. Lysate was clarified by centrifugation at 18,000 *g* for 1 h. The supernatant was filtered (0.45 μm) and purified using glutathione-sepharose as instructed by the manufacturer (Thermo, 16,108).

After purification, proteins were concentrated in a 30 kDa molecular weight cutoff concentrator and buffer exchanged using a Superdex 200 Increase 10/30 Gl column equilibrated in 1X Dulbecco′s Phosphate-Buffered Saline (DPBS) with calcium and magnesium (Sigma, D1283). The protein was > 95% pure, as assessed by SDS-PAGE, and was stored at −80 °C.

### ELISAs

The binding of biotinylated sialoglycans to immobilized GST-SLBRs was performed as described (2, 7, 18). In short, purified SLBRs were diluted to 500 μM in DPBS and added to a 96-well microtiter plate. Plates were incubated overnight at 4 °C. Unbound proteins were removed by aspiration, and wells were rinsed with DPBS. Biotinylated glycans were diluted to the indicated concentrations in DPBS containing 1X Blocking Reagent (Roche, 11,585,762,001) and incubated for 1 h at room temperature. Wells were rinsed three times with DPBS. Streptavidin-conjugated horseradish peroxidase (Sigma, S5512) was added to each well, and the plate was incubated for 1 h at room temperature. The wells were washed twice with DPBS, and then a solution of 0.4 mg *O*-phenylenediamine dihydrochloride (Sigma, P8787) per ml phosphate-citrate buffer (Sigma, P4922) was added to the wells. The absorbance at 450 nm was measured after approximately 20 min. Data were plotted as the means ± standard deviations, with n = 3.

### Far-Western blotting

Far western blotting was performed in duplicate and as previously described (2, 7, 18). Briefly, human or rat plasma was diluted 1:10 into 10 mM Tris-HCl buffer, 1 mM EDTA, pH 8.0, combined with LDS sample buffer and DTT (50 mM final concentration). Samples were boiled for 10 min, and total protein was assessed to standardize gel loading. Glycoproteins were separated by electrophoresis on 3 to 8% polyacrylamide gradient gels (Life Technologies), and then transferred to BioTraceNT (Pall Corporation). Membranes were incubated for 1 h at room temperature with 1× Blocking Reagent in DPBS. GST-SLBRs were then added to a final concentration of 5 nM, and the membranes were incubated for 90 min at room temperature with gentle rocking. After rinsing three times with DPBS, the membranes were incubated for 1 h at room temperature with anti-GST diluted 1:5000 in DPBS containing 1× Blocking Reagent. Membranes were rinsed three times with DPBS and then incubated for 1 h at room temperature with horseradish peroxidase-conjugated goat anti-rabbit antibodies diluted 1:50,000 in DPBS. Membranes were again rinsed three times with DPBS and then developed with SuperSignal West Pico (Thermo Scientific).

## Data availability

Structures and structure factors have been deposited with the Protein Data Bank (PDB, www.pdb.org) under accession codes 9Q5E and 9Q5F. Raw diffraction data are deposited with SBGrid with accession codes 1019 and 1020 and can be accessed at: data.sbgrid.org/dataset/DATAID. Raw data for Far Western Blots are shown in Figure S8.

## Supporting information

This article contains supporting information.

## Conflict of interest

The authors declare that they have no conflicts of interest with the contents of this article.
