# Supplementary material for: Sialic acid identity modulates host tropism of sialoglycan-binding viridans group streptococci
Source: J Biol Chem. 2025 Jul 30;301(9):110540. doi: 10.1016/j.jbc.2025.110540 (PMC12446635; doi:10.1016/j.jbc.2025.110540)
Supplement: Supporting information [file mmc1.pdf]

Sialic Acid Identity Modulates Host Tropism of Sialoglycan-binding Viridans Group Streptococci

KeAndreya M. Morrison[1], Rupesh Agarwal[2], Haley E. Stubbs[3], Hai Yu[4] , Stefan Ruhl[5], Xi Chen[4], Paul M Sullam[6], Barbara A Bensing[6], Jeremy C. Smith[2,7], T. M. Iverson[1,8,*]

[1]Department of Pharmacology, School of Graduate Studies, Meharry Medical College, Nashville, TN, USA
[2]UT/ORNL Center for Molecular Biophysics, Oak Ridge National Laboratory, Oak Ridge, TN, USA
[3]Chemical and Physical Biology Program, Vanderbilt University, Nashville, TN, USA
[4]Department of Chemistry, University of California, Davis, CA, USA
[5]Department of Oral Biology, The State University of New York at Buffalo, Buffalo, NY, USA
[6]Division of Infectious Diseases, Veterans Affairs Medical Center, Department of Medicine, University of California, San Francisco, and the Northern California Institute for Research and Education, San Francisco, CA, USA
[7]Department of Biochemistry and Cellular and Molecular Biology, University of Tennessee, Knoxville, TN

[8]Departments of Pharmacology and Biochemistry, Vanderbilt University, Nashville, TN, USA

*Correspondence to: tina.iverson@vanderbilt.edu

Supporting Information

Contents
Supporting Tables 1 – 2
Supporting Figures 1 – 8

Supporting Table 1

$SLBR_{Hsa}$	32% / 51%	53% / 72%	74% / 85%	78% / 88%	51% / 65%	
$SLBR_{SrpA}$	30% / 47%	53% / 70%	54% / 67%	56% / 65%		
$SLBR_{UB10712}$	33% / 51%	52% / 72%	77% / 89%			
$SLBR_{SK678}$	31% / 50%	51% / 68%				
$SLBR_{SK1a}$	37% / 52%					
	$SLBR_{GspB}$	$SLBR_{SK1a}$	$SLBR_{SK678}$	$SLBR_{UB10712}$	$SLBR_{SrpA}$	$SLBR_{Hsa}$

Supporting Table 1 Sequence identity and similarity of SLBRs used in the study.

Supporting Table 2

$SLBR_{Hsa}$	0.744 Å	0.749 Å	0.517 Å	0.333 Å	0.989 Å	
$SLBR_{SrpA}$	1.037 Å	0.719 Å	0.853 Å	0.895 Å		
$SLBR_{UB10712}$	0.703 Å	0.759 Å	0.319 Å			
$SLBR_{SK678}$	0.703 Å	0.820 Å				
$SLBR_{SK1a}$	0.886 Å					
	$SLBR_{GspB}$	$SLBR_{SK1a}$	$SLBR_{SK678}$	$SLBR_{UB10712}$	$SLBR_{SrpA}$	$SLBR_{Hsa}$

Supporting Table 2 Pairwise RMSD values of C_α atoms for structures of SLBR Siglec domains used in this study. $SLBR_{Hsa}$ (PDB 6EFC (18)), $SLBR_{SrpA}$ (PDB 5EQ3 (36)), $SLBR_{UB19712}$ (PDB 6EFC(18)), $SLBR_{GspB}$ (PDB 6EFA (16), $SLBR_{SK1}$ (PDB 6VS7(37)), and $SLBR_{SK678}$ (PDB 6EFI (18)).

Supporting Figure 1

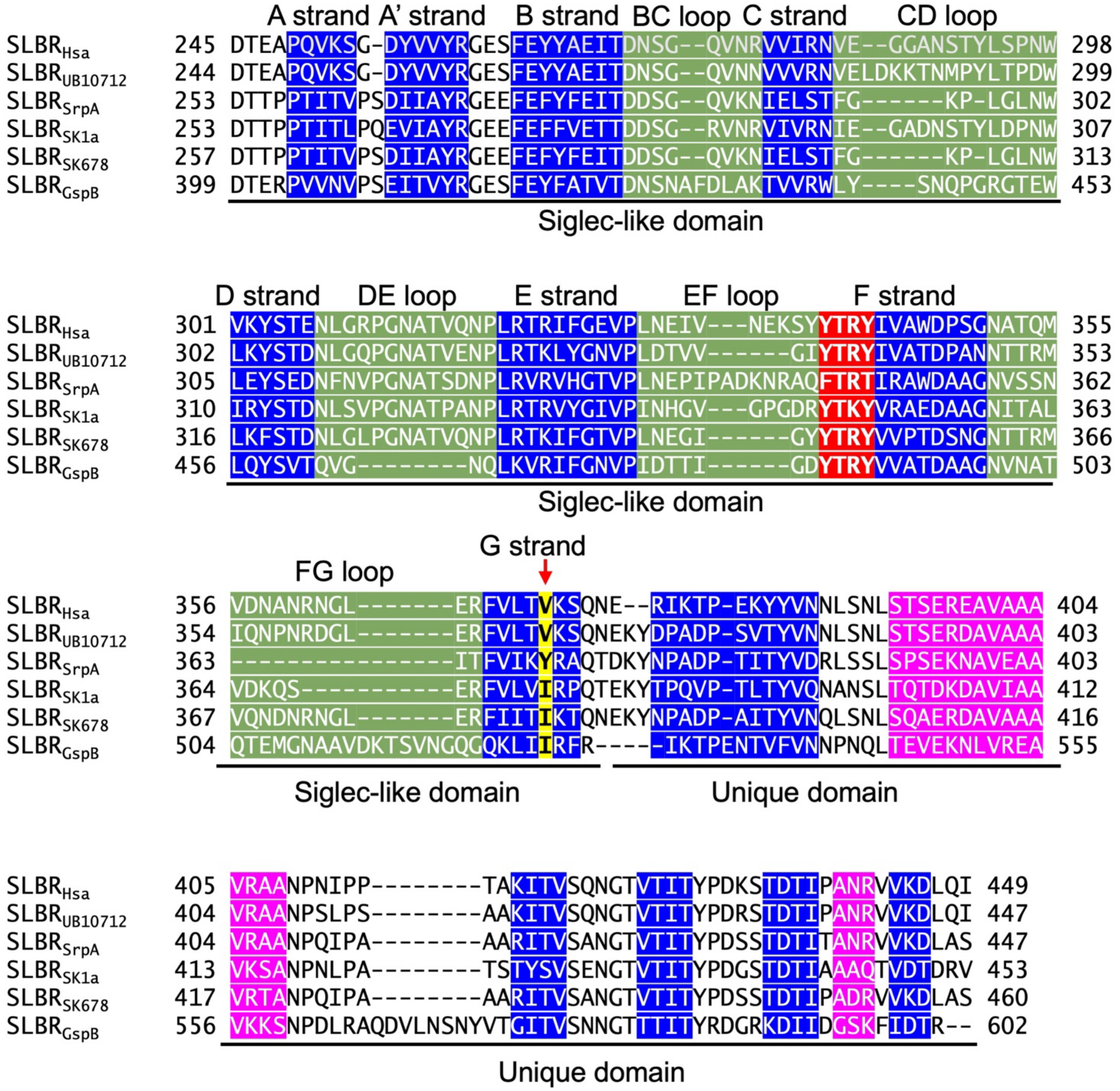

Supporting Figure 1 Sequence alignment of select SLBRs with characterized sialoglycan preferences. Structural elements in the V-set Ig fold are marked. β-strands are highlighted with blue backgrounds, α-helices are highlighted with pink backgrounds, interstrand loops are highlighted with green backgrounds, the Φ-T-R-*x* sequence motif is highlighted with a red background, the residues equivalent to $SLBR_{Hsa}^{V370}$ and $SLBR_{SrpA}^{Y368}$ in the G strand are highlighted with a yellow background. Sequences are from WP_081102781.1 from *S. gordonii* strain Challis ($SLBR_{Hsa}$) (14, 18), WP_045635027.1 from *S. gordonii* strain UB10712 (18, 58), WP_011836739.1 from *S. sanguinis* strain SK36 ($SLBR_{SrpA}$) (36, 59), WP_080555651.1 from *S. sanguinis* strain SK1 ($SLBR_{SK1a}$ and $SLBR_{SK1b}$) (37, 60), WP_125444035.1 from *S. sanguinis* strain SK678 (18, 60), and WP_125444382.1 from *S. gordonii* strain M99 ($SLBR_{GspB}$) (16, 61). Pairwise sequence identity/similarity is summarized in **Supporting Table 1** and pairwise RMSD values of C_α atoms for the Siglec domains are summarized in **Supporting Table 2.**

Supporting Figure 2

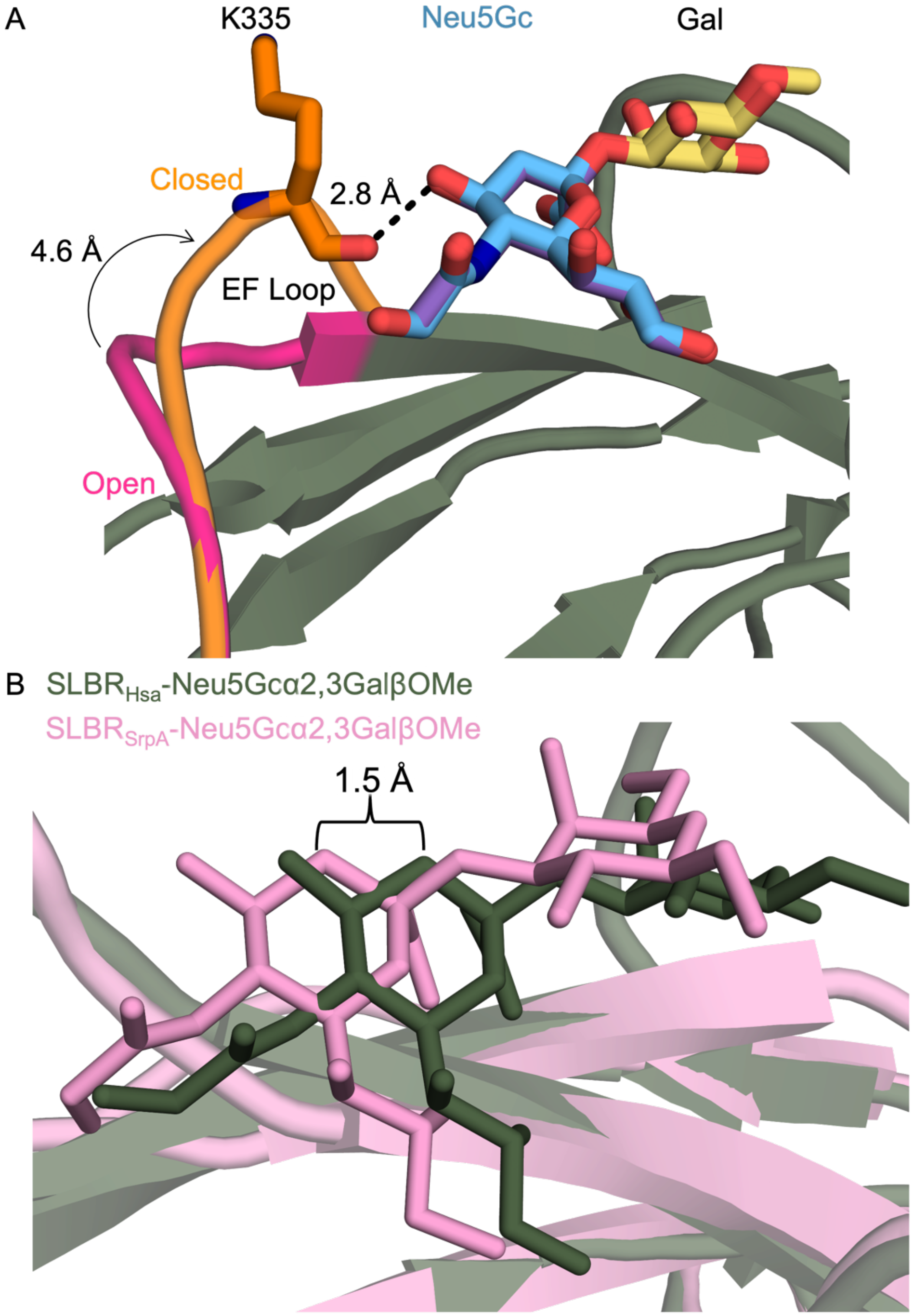

Supporting Figure 2 Features of sialyldisaccharide-bound SLBR$_{Hsa}$ and SLBR$_{SrpA}$. *A,* In X-ray crystal structures of SLBR$_{Hsa}$-Neu5Gc, a hydrogen bond forms between SLBR$_{Hsa}^{K335}$ and O4 of Neu5Gc or Neu5Ac. The open position bound in the unliganded structure (PDB 6EFC (18)) is shown for comparison. *B,* In SLBR$_{SrpA}$-Neu5Gc (PDB 5EQ3 (36)) and SLBR$_{Hsa}$–Neu5Gc, the disaccharide binds above the F strand but has a lateral shift in position of 1.5 Å with respect to the ΦTRX motif.

Supporting Figure 3

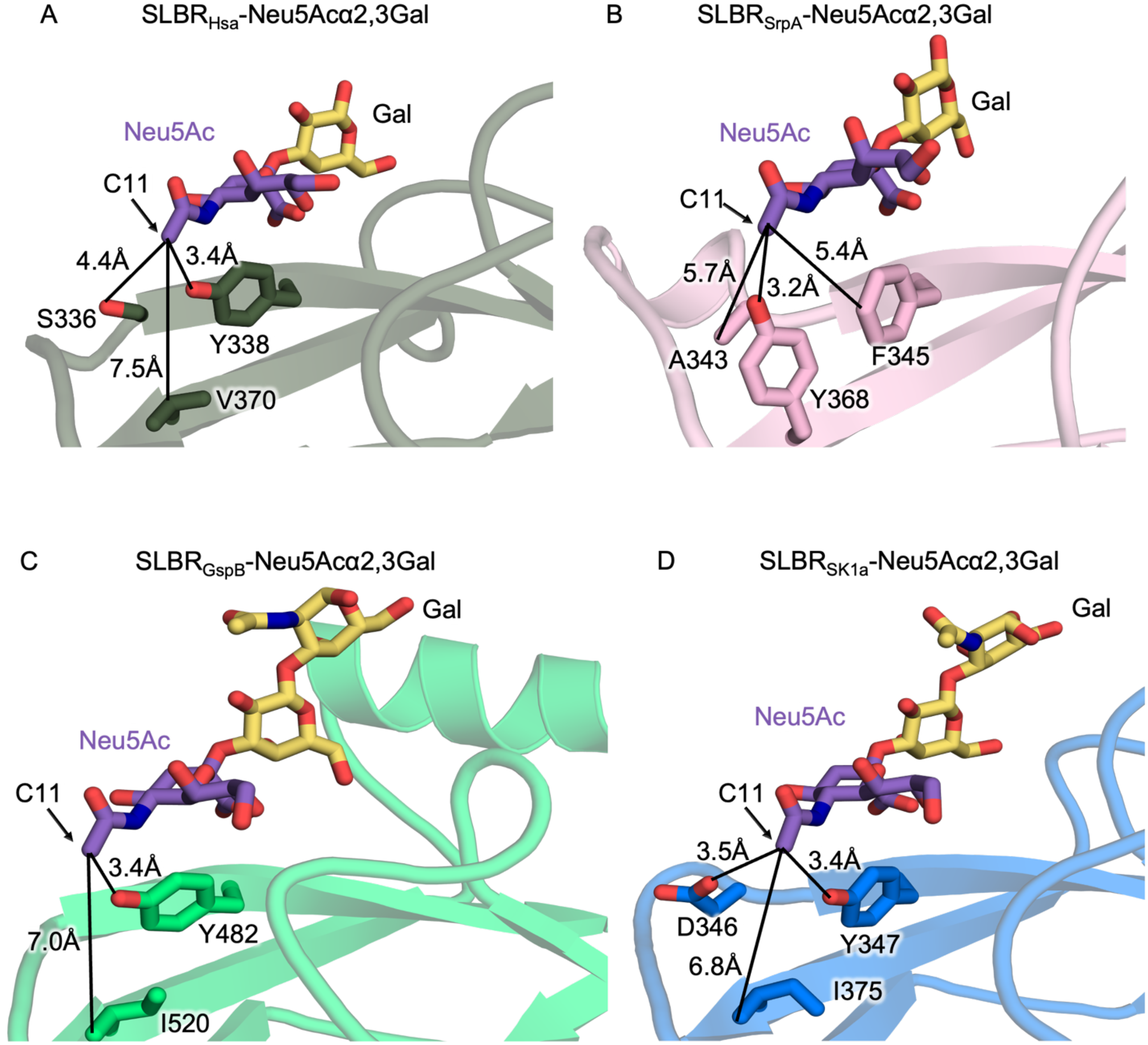

Supporting Figure 3 Comparison of SLBRs bound to sialoglycans terminated with Neu5Ac. *A,* SLBR$_{Hsa}$, *B,* SLBR$_{SrpA}$ (PDB 5EQ3 (36)), *C,* SLBR$_{GspB}$ (PDB 6EFA (16)) and *D,* SLBR$_{SK1a}$ (PDB 6VS7 (37)). Distances between adjacent atoms are shown with solid black lines.

Supporting Figure 4

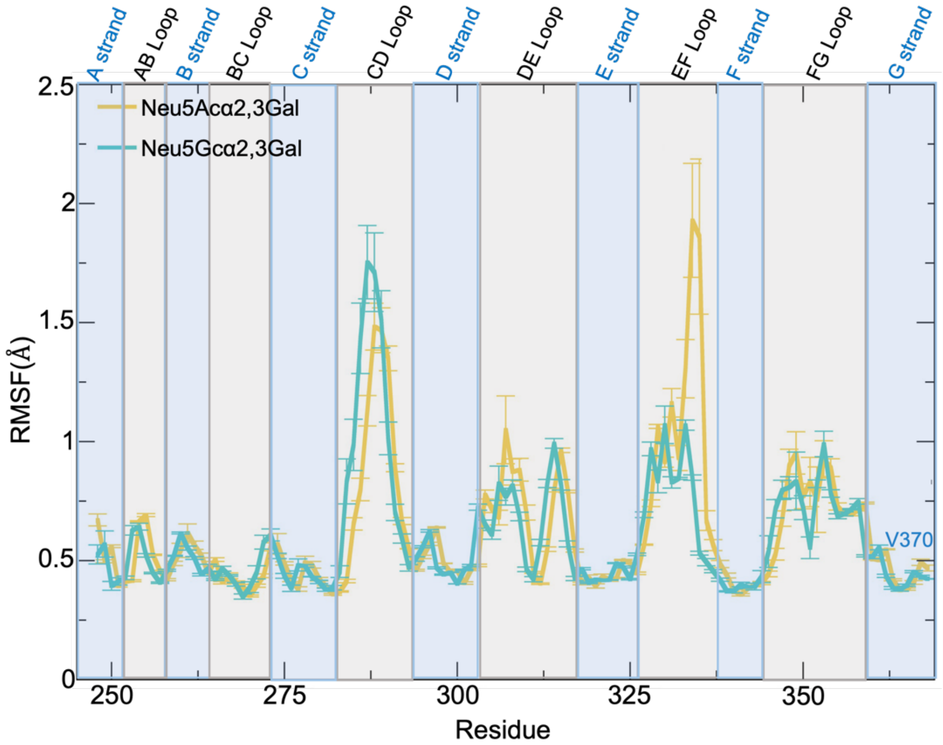

Supporting Figure 4 RMSF values of the Siglec-like domain of $SLBR_{Hsa}$–Neu5Gc and $SLBR_{Hsa}$–Neu5Ac. RMSF plot for the Siglec-like domain of $SLBR_{Hsa}$ during MD simulations show backbone flexibility in the presence of Neu5Ac (*gold*) or Neu5Gc (*cyan*). Regions corresponding to the interstrand loops are highlighted with *grey* backgrounds and β-stands of the V-set Ig fold are highlighted with *blue* backgrounds.

Supporting Figure 5

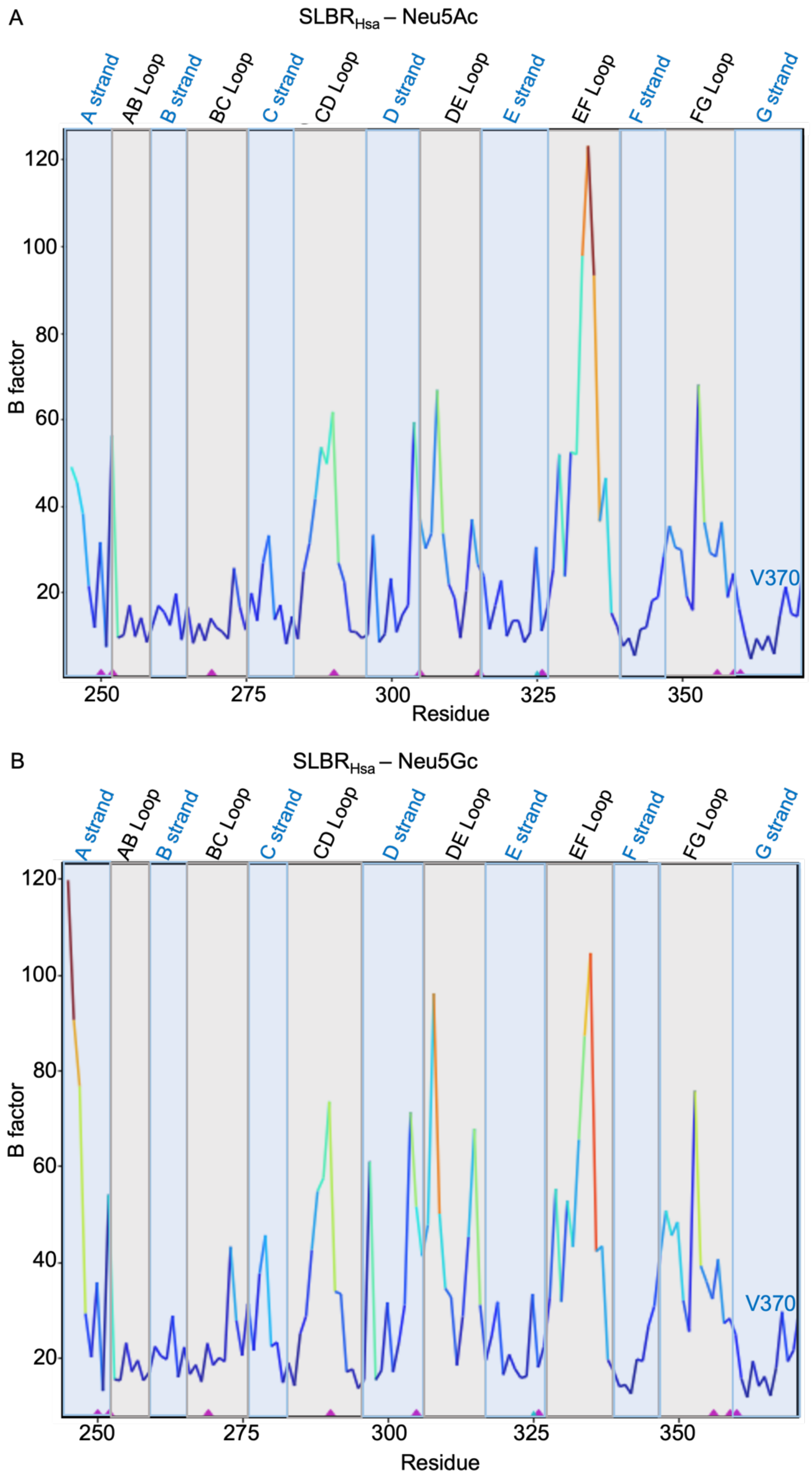

Supporting Figure 5 Crystallographic temperature factors of the Siglec-like domain of SLBR$_{Hsa}$. *A,* SLBR$_{Hsa}$–Neu5Ac and *B,* SLBR$_{Hsa}$–Neu5Gc. Residues are colored by B-factor magnitude, with warmer colors representing a higher temperature factor. Regions corresponding to the interstrand loops are highlighted with *grey* backgrounds and β-stands of the V-set Ig fold are highlighted with *blue* backgrounds.

Supporting Figure 6

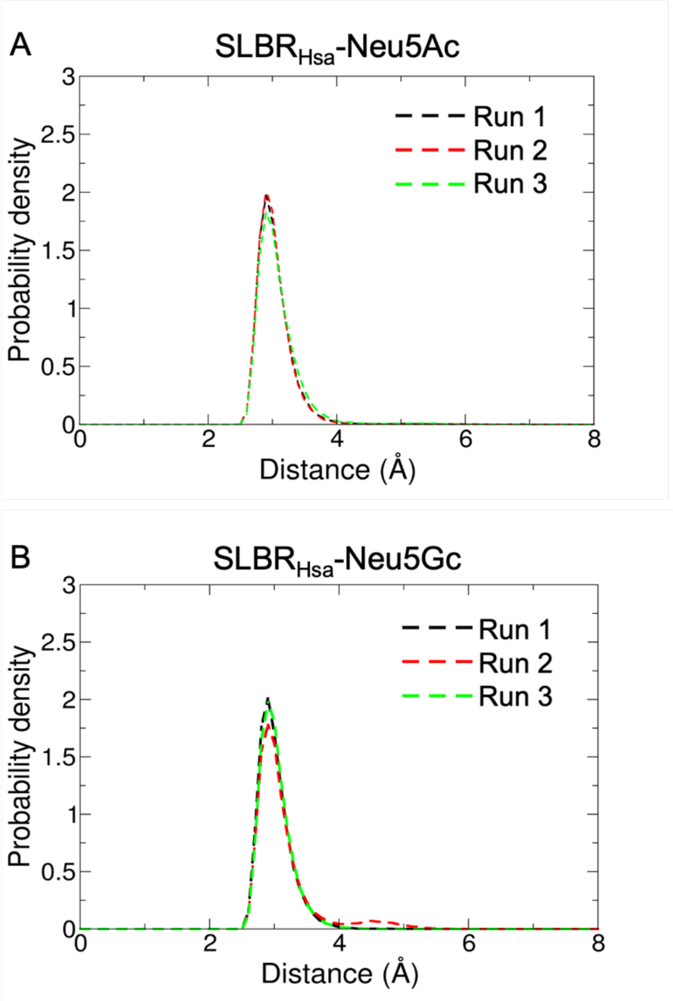

Supporting Figure 6 Probability distributions of the distance between the SLBR$_{Hsa}$^{K335} backbone carbonyl and Neu5Gc-O4 in simulations. The simulations used to disaccharides terminated in *A,* Neu5Ac or *B,* Neu5Gc. The ~3 Å distance is consistent with a hydrogen-bonding interaction. All simulations were performed in triplicate.

Supporting Figure 7

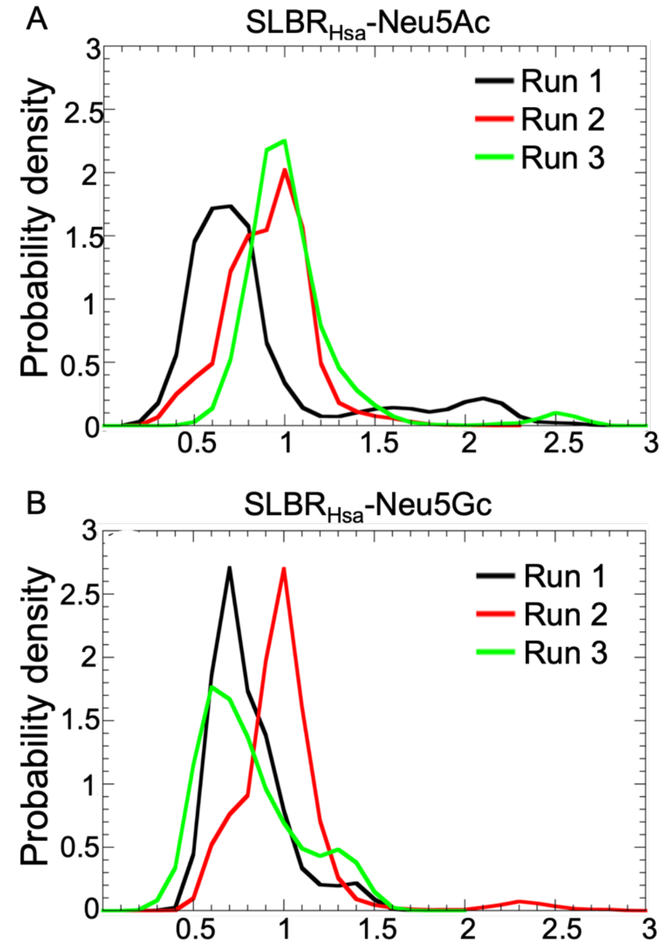

Supporting Figure 7 Probability distribution of RMSD values of disaccharides bound to $\mathbf{SLBR_{Hsa}}$. *A,* Neu5Acα2,3-Gal and *B,* Neu5Gcα2,3-Gal. Three independent molecular dynamics simulations were performed. Mean RMSD Neu5Acα2,3-Gal = 0.85 Å ($\pm$0.14 Å). Mean RMSD Neu5Gcα2,3-Gal = 0.83 Å ($\pm$0.15 Å).

Supporting Figure 8

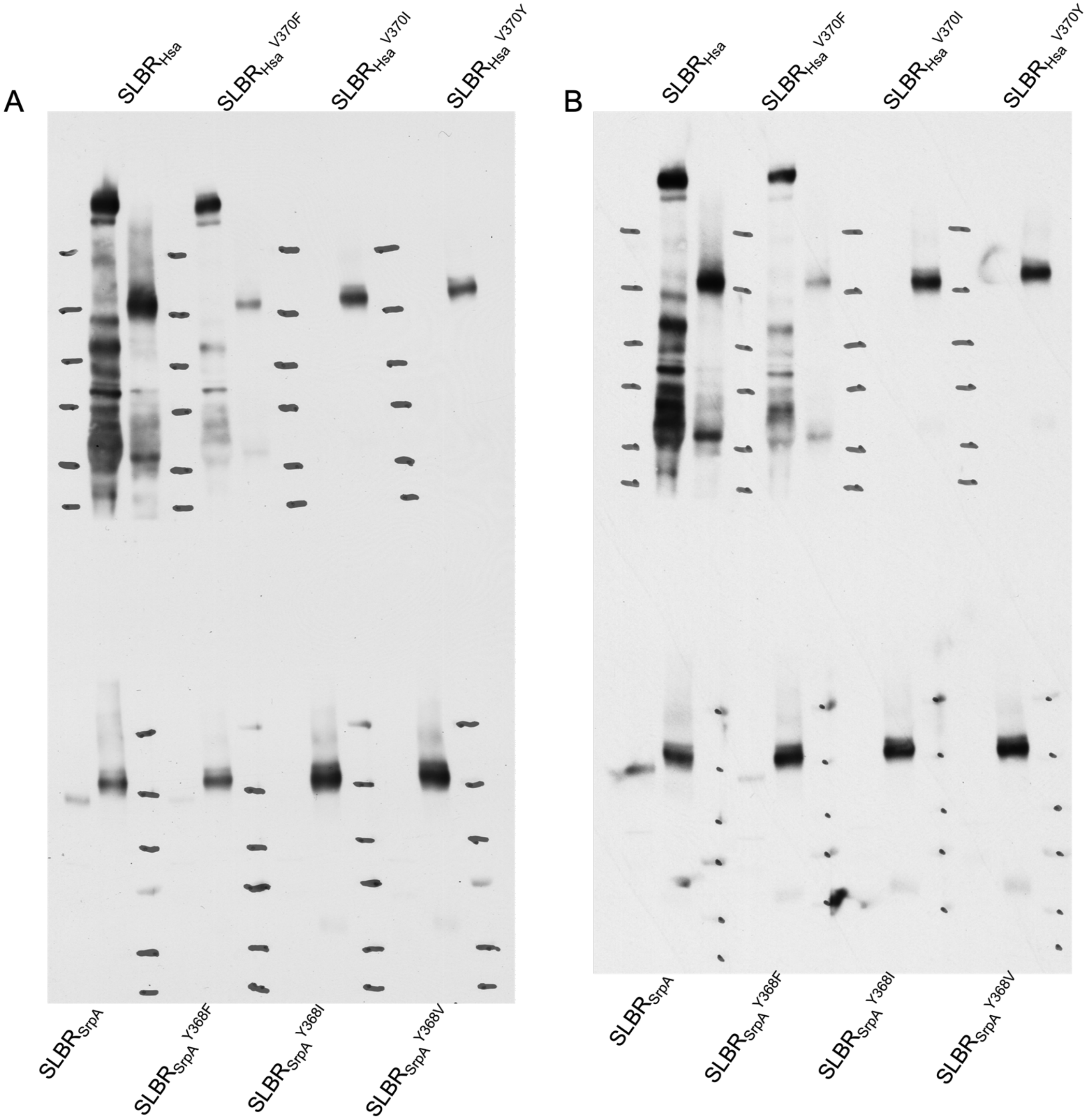

Supporting Figure 8 Uncropped far Western blots of SLBR binding to human or rat plasma glycoproteins. *A,* Far-Western blots of wild-type and mutant GST-SLBR$_{Hsa}$ (top panels) and GST-SLBR$_{SrpA}$ (bottom panels) against plasma glycoproteins. Glycoproteins were separated by electrophoresis through a 3–8% polyacrylamide gradient transferred to membranes, exposed to each GST-SLBR, then probed with an anti-GST primary antibody. *B,* A replicate performed on a separate day. Hand-drawn lines between SLBR variants identify the positions of the molecular weight markers.
